# Supplementary material for: How does a small molecule bind at a cryptic binding site?
Source: PLoS Comput Biol. 2022 Mar 3;18(3):e1009817. doi: 10.1371/journal.pcbi.1009817 (PMC8893328; doi:10.1371/journal.pcbi.1009817)
Supplement: S1 Text — Table A, Simulation details; Fig A, Conformational fluctuation with respect to the average; Fig B, Interaction energy of SP4206 with IL2 in simulations; Fig C, Comparison of unbound and ligand-bound conformations of IL2. (PDF) [file pcbi.1009817.s001.pdf]

Supporting Information

How does a small molecule bind at a cryptic binding site?

Supporting Table and Figures

Table A. Simulation details

	Sim #	Length (µs)	Protein	Small molecule(s) (S.M.)	# copies of S.M.	Specific Binding	Binding time range (µs)
SP4206	1	31.0	IL2	SP4206	3	yes	0.25—
	2	21.0	IL2	SP4206	3	no	
	3	18.0	IL2	SP4206	1	yes	0.1—
	4	25.0	IL2	SP4206	1	no	
	5	22.0	IL2	SP4206	1	no	
	6	17.0	IL2	SP4206	1	no	
	7	10.0	IL2	SP4206	1	no	
	8	10.0	IL2	SP4206	1	yes	1.05—
	9	10.0	IL2	SP4206	1	no	
	10	10.0	IL2	SP4206	1	yes	0.43—
	11	10.0	IL2	SP4206	1	no	
	12	10.0	IL2	SP4206	1	yes	
	13	10.0	IL2	SP4206	1	yes	0.62—
	14	10.0	IL2	SP4206	1	no	
	15	10.0	IL2	SP4206	1	yes	8.0—
	16	10.0	IL2	SP4206	1	no	
SP4206 analogs	17	20.0	IL2	SP4206-1	2	yes	16—
	18	20.0	IL2	SP4206-1	2	yes	0.1—
	19	20.0	IL2	SP4206-2	2	yes	1—
	20	20.0	IL2	SP4206-2	2	yes	0.6—
	21	20.0	IL2	SP4206-3	2	no	
	22	20.0	IL2	SP4206-3	2	yes	15—
	23	20.0	IL2	SP4206-3	2	no	
	24	20.0	IL2	SP4206-3	2	yes	6.1—
fragments	25	24.0	IL2	Fragments S, T	2	no	
	26	53.9	IL2	Fragments S, T	1	yes	T(23.6—48.4)
	27	37.0	IL2	Fragments N, M, L	1	yes	M(0.7—24.1),
	28	16.2	IL2	Fragments N, Q, R, P, L	2	no	

	29	16.5	IL2	Set of 18 fragments	1	no	
Compound 43a	30	30.9	Bcl-xL	Compound 43a	1	no	
	31	36.0	Bcl-xL	Compound 43a	1	no	
	32	13.0	Bcl-xL	Compound 43a	1	no	
	32	38.0	Bcl-xL	Compound 43a	3	yes	31—
	34	18.9	Bcl-xL	Compound 43a	3	no	
	35	11.7	Bcl-xL	Compound 43a	3	yes	3.1—

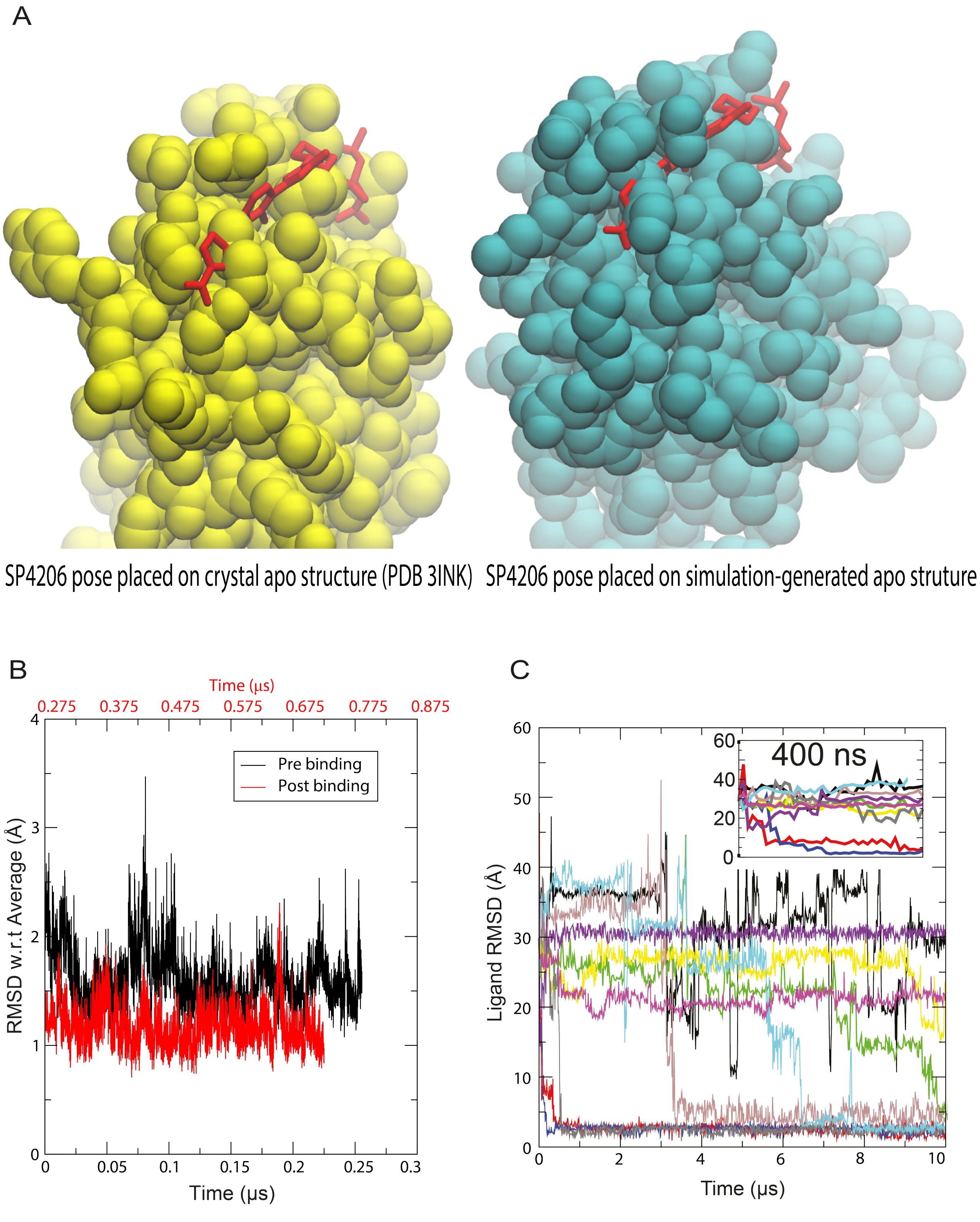

Fig A. Conformational fluctuation with respect to the average. (A) Left panel, the native

binding pose of SP4206 superimposed on apo IL2 crystal structure 3INK; right panel; the native binding pose of SP4206 superimposed on an IL2 conformation generated prior to the binding event in an IL2-SP4206 binding simulation. (In this simulation, the binding groove later emerged in the presence of SP4206, and SP4206 arrived at the native binding pose.) (B) This figure is a supplement to Fig 1D. The RMSD of the residues forming the binding groove (residues 61–65, 54, 55, 58, 59, 82, 85, 88, 92, 131) with respect to the average conformation of the period (pre-binding: 0–0.25 μs; post binding: 0.275–0.5 μs) is shown. As shown, the conformational fluctuation was reduced after SP4206 binding. (C) The time series of SP4206 RMSD with respect to the crystal binding pose in 10 additional simulations in which SP4206 was placed in a random initial position away from the protein. As shown, four simulations arrived at the native binding pose and two arrived at poses similar to the native. The inset is a magnification of the first 400 ns of the simulation.

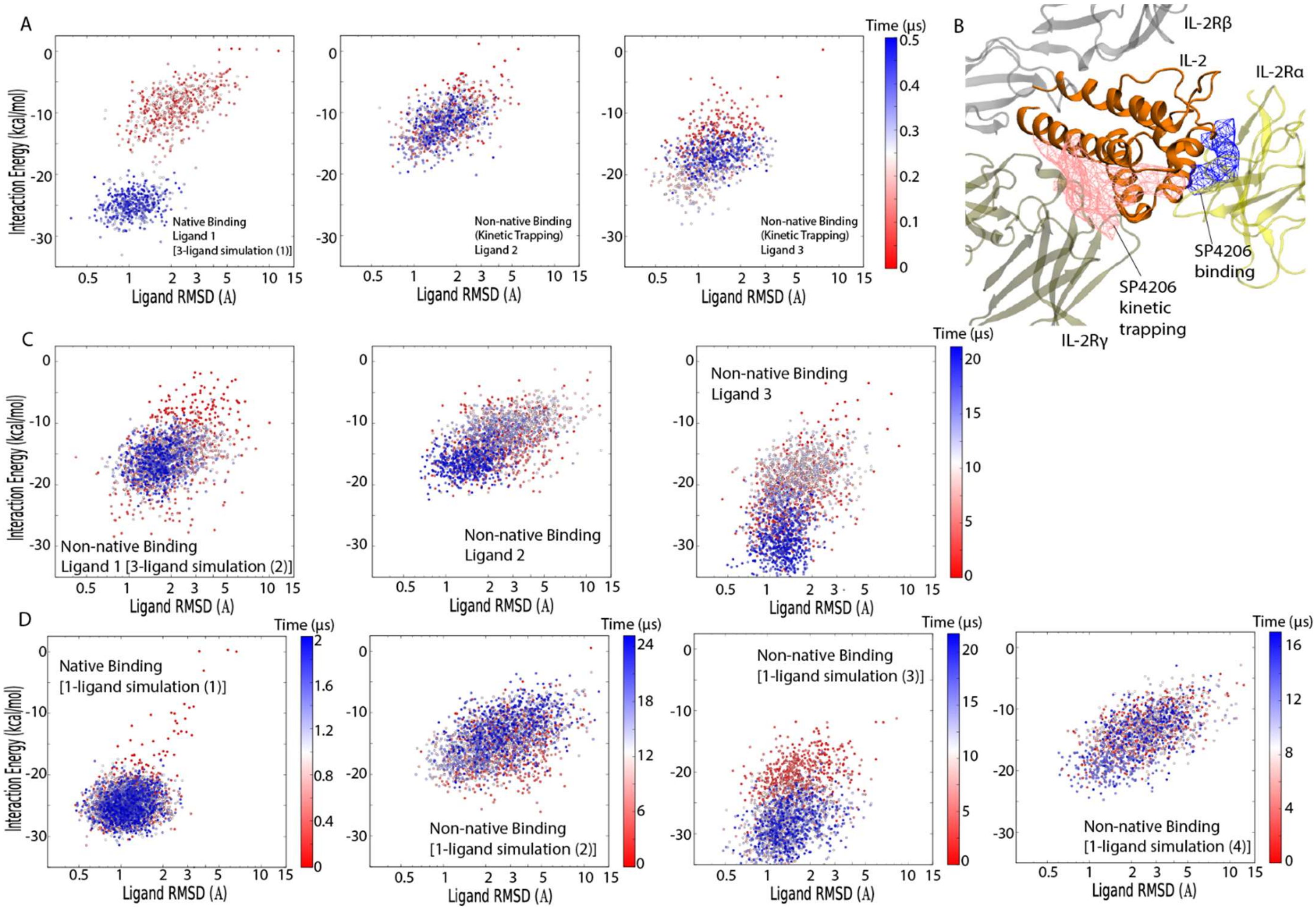

Fig B. Interaction energy of SP4206 with IL2 in simulations. The estimated ligand-protein interaction energy (calculated with the GBSA method) and ligand conformational fluctuation were analyzed for six simulations. (A) A three-ligand simulation, in which one of the three ligands reached the native binding pose at 0.25 μs (Ligand 1), mapped to a 2D space of energy and ligand conformational fluctuation as measured by RMSD with respect to the conformation of the previous time step (*x*-axis). (B) The receptor domains (IL2Rα, β, and γ) bound with IL2 are shown with meshes indicating the high-occupancy regions for SP4206. (C) Another three-ligand simulation, in which none of the ligands reached the native pose. (D) Four one-ligand simulations. The ligand reached the native binding pose quickly (at 0.1 μs) in the first

simulation, and as a result, the pre-binding dissociated state was under-sampled. In the other three simulations, the ligand did not reach the native pose.

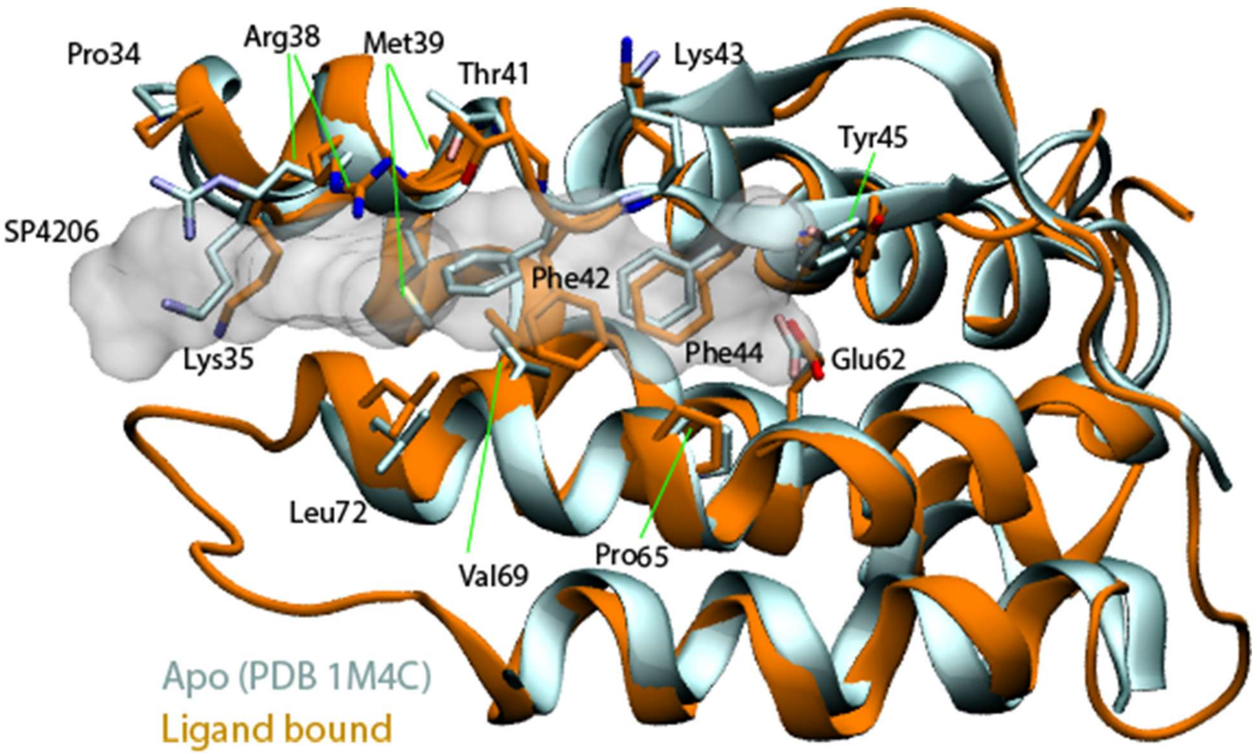

Fig C. Comparison of unbound and ligand-bound conformations of IL2. The backbone ribbon of each of the two conformations is rendered, along with the residues in contact with the bound ligand, which are rendered as a transparent surface. The primary difference between the two conformations involves a side-chain rearrangement of the residues that form the binding site, particularly Arg38, Met39, and Phe42.
